# Including undocumented migrants in universal health coverage: a maternal health case study from the Thailand-Myanmar border

**DOI:** 10.1186/s12913-021-07325-z

**Published:** 2021-12-07

**Authors:** Naomi Tschirhart, Wichuda Jiraporncharoen, Rojanasak Thongkhamcharoen, Kulyapa Yoonut, Trygve Ottersen, Chaisiri Angkurawaranon

**Affiliations:** 1 Oslo Group on Global Health Policy, Department of Community Medicine and Global Health and Centre for Global Health, Institute of Health and Society, Faculty of Medicine, University of Oslo, Oslo, Norway; 2 Interdisciplinary School of Health Sciences, Department of Health Sciences, University of Ottawa, Ottawa, Canada; 3 Department of Family Medicine, Chiang Mai University, Chiang Mai, Thailand; 4 Department of Social Medicine, Maesot General Hospital, Sripanich Road, Maesot, Tak Thailand; 5 Division for Health Services, Norwegian Institute of Public Health, Oslo, Norway

**Keywords:** Universal health coverage, Health insurance, Migrant, Undocumented, Maternal health, Emergency obstetrical care, Labor

## Abstract

**Background:**

Many countries aspiring to achieve universal health coverage struggle with how to ensure health coverage for undocumented migrants. Using a case study of maternal health care in a Thailand-Myanmar border region this article explores coverage for migrants, service provision challenges and the contribution of a voluntary health insurance program.

**Methods:**

In 2018 we interviewed 18 key informants who provided, oversaw or contributed to maternal healthcare services for migrant women in the border region of Tak province, Thailand.

**Results:**

In this region, we found that public and non-profit providers helped increase healthcare coverage beyond undocumented migrants’ official entitlements. Interview participants explained that Free and low-cost antenatal care (ANC) is provided to undocumented migrants through migrant specific clinics, outreach programs and health posts. Hospitals offer emergency birth care, although uninsured migrant patients are subsequently billed for the services. Care providers identified sustainability, institutional debt from unpaid obstetric hospital bills, cross border logistical difficulties and the late arrival of patients requiring emergency lifesaving interventions as challenges when providing care to undocumented migrants. An insurance fund was developed to provide coverage for costly emergency interventions at Thai government hospitals. The insurance fund, along with existing free and low-cost services, helped increase population coverage, range of services and financial protection for undocumented migrants.

**Conclusions:**

This case study offers considerations for extending health coverage to undocumented populations. Non-profit insurance funds can help to improve healthcare entitlements, provide financial protection and reduce service providers’ debt. However, there are limits to programs that offer voluntary coverage for undocumented migrants. High costs associated with emergency interventions along with gaps in insurance coverage challenge the sustainability for NGO, non-profit and government health providers and may be financially disastrous for patients. Finally, in international border regions with high mobility, it may be valuable to implement and strengthen cross border referrals and health insurance for migrants.

**Supplementary Information:**

The online version contains supplementary material available at 10.1186/s12913-021-07325-z.

## Background

Universal health coverage (UHC), inclusive of financial protection and access to quality health services, is a guiding global health objective as identified in Sustainable Development Goal (SDG) 3.8 [1]. However, almost every country aspiring or claiming to achieve UHC struggles with how to include undocumented migrants. Persons with irregular status fall outside of the clear delineated categories of citizens and registered migrant workers and present challenges for governments on how to fund and ensure health coverage for these populations. In theory, the universal and inclusive nature of UHC positions it as a goal which should be inclusive of migrants regardless of administrative status. However, in practice access to healthcare for migrants around the globe is often linked to legal status and associated entitlements, and undocumented migrants often have limited entitlements and challenges accessing appropriate care [2–4]. For countries that attempt to include migrants in universal health coverage there are different financing mechanisms such as general tax based financing or a self-financed pool with voluntary contributions [3]. Extending UHC to include undocumented persons furthers the right to health, that everyone should be able to access the services they need regardless of their migration status [5].

Maternal healthcare, inclusive of emergency interventions during childbirth, is often considered as a basic component of UHC service packages in low and middle-income countries (LMICs). The “proportion of births attended by skilled health personnel” is also a key indicator for achieving SDG targets to reduce maternal mortality [6]. Access to appropriate care, inclusive of caesarean sections where necessary, is imperative for women experiencing complications during childbirth and can be live saving for women and babies. In countries where undocumented migrants do not have legal entitlement to affordable public healthcare, pregnant migrant women may be billed directly for life saving surgeries and occur associated unsurmountable debt. Catastrophic health expenditures, where out of pocket payments for healthcare are unaffordable, also occurs in countries where citizens do not have sufficient health insurance coverage. A study in Myanmar reported catastrophic healthcare expenditures among 37% of households when using 10% of total expenditure as the threshold [6]. Rates of catastrophic expenditure were higher among those with low incomes, living in a rural area and households whose members experienced hospitalization [7].

Coverage for undocumented migrants remains a challenge for the realization of UHC, and there are very few examples in the literature from LMICs of care provision to undocumented migrants, particularly from providers’ perspectives. UHC maternal health indicators measured at the national level also may obscure the outcomes of subgroups including undocumented migrant women who face additional difficulties receiving EmOC comparative to the Thai population [8, 9]. In this article we use a case study of maternal healthcare for migrants in a border region of Thailand to explore inclusion of undocumented migrants in UHC.

Thailand is internationally recognized as a leader in both UHC and the development of mechanisms to provide healthcare coverage for migrants [2, 10–13]. Throughout the country, entitlement to healthcare from the Thai public system is linked to legal status. Migrants who are documented and have the appropriate paperwork can enroll in the government social security scheme (SSS) which is funded through employee, employer and government contributions [14]. Migrants who are not able to join the SSS can join the Migrant Health Insurance Scheme (MHIS), which is self-funded through migrant contributions [14]. Recent policy updates necessitate that MHIS applicants also become documented and get a work permit before purchasing coverage [15]. In response to the unmet needs of undocumented migrants, an NGO developed a The Migrant Fund, a non-profit health insurance scheme for this population [16]. The Migrant Fund is a donor and contributor financed scheme that aims to become fully self-financed in the future [16].

Mae Sot city and surrounding areas on the Thai side of the Thailand-Myanmar border are an area which have historically hosted a large number of migrants and refugees from nearby Myanmar and have a tradition of NGO, non-profit and government healthcare provision for migrants. Today the border remains fluid and patients come from both the Thai countryside and city as well as from Myanmar to seek care. At the time of data collection, NGO funding for migrant healthcare in the border region was decreasing which influenced the development of the Migrant Fund (M-FUND) and other collaborative initiatives between service providers who provide healthcare to migrants. The M-FUND was launched in 2017 and the program was being updated in 2018 when we collected our data [16]. All of the changes to health services for migrants occurred in the larger context of a reduction of funding in refugee camps along the Thailand-Myanmar border and a steep decline in refugee health services that undocumented migrants would have previously been able to access [17]. The top three infectious diseases (malaria, tuberculosis and HIV) fall under the Global Fund initiative and have regional strategies to prevent spread and improve control and associated funding with an awareness of migrant worker flows across borders, but Maternal and Child Health (MCH) falls outside their mandates [18, 19].

The objective overarching this qualitative study was to explore service provision of emergency obstetric care in labor (EmOC) for documented and undocumented migrant women and to identify health systems adaptations that improve access. In this paper we detail how maternal migrant healthcare coverage is provided to migrants in a Thai border region, discuss challenges associated with providing healthcare to undocumented migrants from a clinician perspective and highlight the contribution of a voluntary health insurance program. We present this as a case study of extending healthcare coverage to undocumented pregnant migrants.

## Methods

In May and June 2018, we held 18 individual interviews with key informants in Tak province, Thailand close to the Thailand-Myanmar border. Prior to beginning recruitment we identified clinics, hospitals and organizations that provide maternal care to migrant patients in the region. Team members then directly contacted individuals who had experience providing, overseeing or contributing to maternal healthcare services for pregnant migrant women and invited them to participate in an interview. Five administrators, eleven doctors, nurses, medics and health workers and two social service personnel participated in the interviews. Before beginning interviews, we explained the nature of the project to participants and collected written consent. Two authors (WJ and CA) conducted interviews in Thai and NT interviewed participants in English and in Karen with assistance from an interpreter. Interviews lasted approximately 45 min. Using a semi-structured interview guide (supplementary file 1), we asked participants about the antenatal and emergency birth services that are available to migrant women, who is entitled to this care, care accessibility as well as health system capacity, financing and sustainability. We had developed the interview guide through an iterative process by incorporating feedback from team members who are Thai clinicians as well as stakeholders who support migrant healthcare. This engagement helped us to ensure the questions would be appropriate and well understood by participants.

We had the audio files transcribed, translated into English and uploaded into NVivo software. Using thematic analysis NT coded the data for predetermined and emergent themes related to healthcare coverage and service provision challenges [20]. NT met with WJ, CA to discuss preliminary findings relevant to care provision and met with TO, to review results in the context of UHC. All authors contributed to the manuscript.

Our project was reviewed and approved by Research Ethics Committee 2 at the Faculty of Medicine, Chiang Mai University (FAM-2560-05204). The project was also reviewed and approved through the standard internal process at the Department of Community Medicine and Global Health at the University of Oslo and notified to the Data Protection Official for Research at NSD - Norwegian Centre for Research Data (58542). We also received feedback from the Tak Province Community Advisory Board. Written informed consent was obtained from all participants.

## Results

### Maternal health coverage for migrants in a Thai border region

During the interviews research participants explained that in Tak province’s Thai border area healthcare for migrants, including maternal healthcare, is provided by both Thai government and non-profit providers (see Table 1). Free and low-cost antenatal care is available through a number of sources including village healthcare centres run by the Thai healthcare system, community health posts where care is provided by foreign health volunteers with support from Thai healthcare providers and NGO/non-profit clinics that provide care specifically for documented and undocumented migrants. Several Thai hospitals also provide outreach services to reach populations in rural areas. We observe that this hybrid public and non-profit healthcare environment has helped stretch healthcare coverage beyond migrants’ entitlements as articulated in National Thai policies.

**Table 1:** Antenatal care providers who work with migrant populations along the Thailand-Myanmar border and services provided

Type	Provider	Services	Cost
Health promoting hospitals	Thai government	Antenatal care (ANC) check ups	Free
Community health posts	Thaigovernment /NGO	ANC	Free
Migrant specific clinics	NGO or non-profit	ANC, ultrasound, childbirth, emergency delivery, emergency vacuum, infant resuscitation	Free or low-cost
Thai district hospitals	Thai government	ANC, ultrasound, childbirth, emergency delivery, emergency vacuum, infant resuscitation, caesarean section	Fee for service, Insurance coverage available
Thai regional hospital	Thai government	ANC, ultrasound, childbirth, caesarean section, emergency delivery, emergency vacuum, infant resuscitation	Fee for service, Insurance coverage available
Hospital in Myanmar border city	Myanmar government	ANC, ultrasound, childbirth birth, emergency delivery, emergency vacuum, infant resuscitation caesarean section	Fee for service

Migrants who are documented and have insurance coverage through the SSS or MHIS schemes can access antenatal care and emergency birth care through the Thai government hospital with which they are affiliated. Women with this insurance must pay premiums but will have to pay only 30 baht (1 USD) to use services. However, there are restrictions on where the services for these policies can be utilized and the insurance is linked to specific hospitals upon registration. This causes challenges for migrants who purchased insurance in one geographic region and move to another as the insurance coverage is only valid in the first jurisdiction. The premium for pregnancy is paid in a lump sum which is frequently not possible to obtain for daily wage earners as compared to the MFUND which offers monthly payments.

For migrants who are undocumented, the situation is more complex and while low cost or free antenatal care is available for this group from a few sources, those without insurance coverage would be requested to pay in full for emergency care received at a Thai hospital. Undocumented migrants now have the option to purchase coverage from the M-FUND at a cost of 100 baht a month (3 USD), which would cover expenses at the Thai government hospital if they need to be referred for emergency birth services. The M-FUND increases the amount of financial protection offered to undocumented migrants who require expensive treatments such as caesarean sections which cost approximately 15–20,000 thb (depending on the baby) 480 to 650 US dollars at the local Thai hospital. A key informant from a clinic explains:

“ANC is totally free, and for some people who don’t sign up for M-Fund they still get free ANC and delivery care if it’s uncomplicated. But for example, last night we had our first non M-Fund referral which just feels awful because you just want to give them this service and take the stress (of associated medical bills) away”.

Pregnant women must register and pay into the M-FUND for 14 days before they are eligible to use the coverage.

**Fig. 1:**
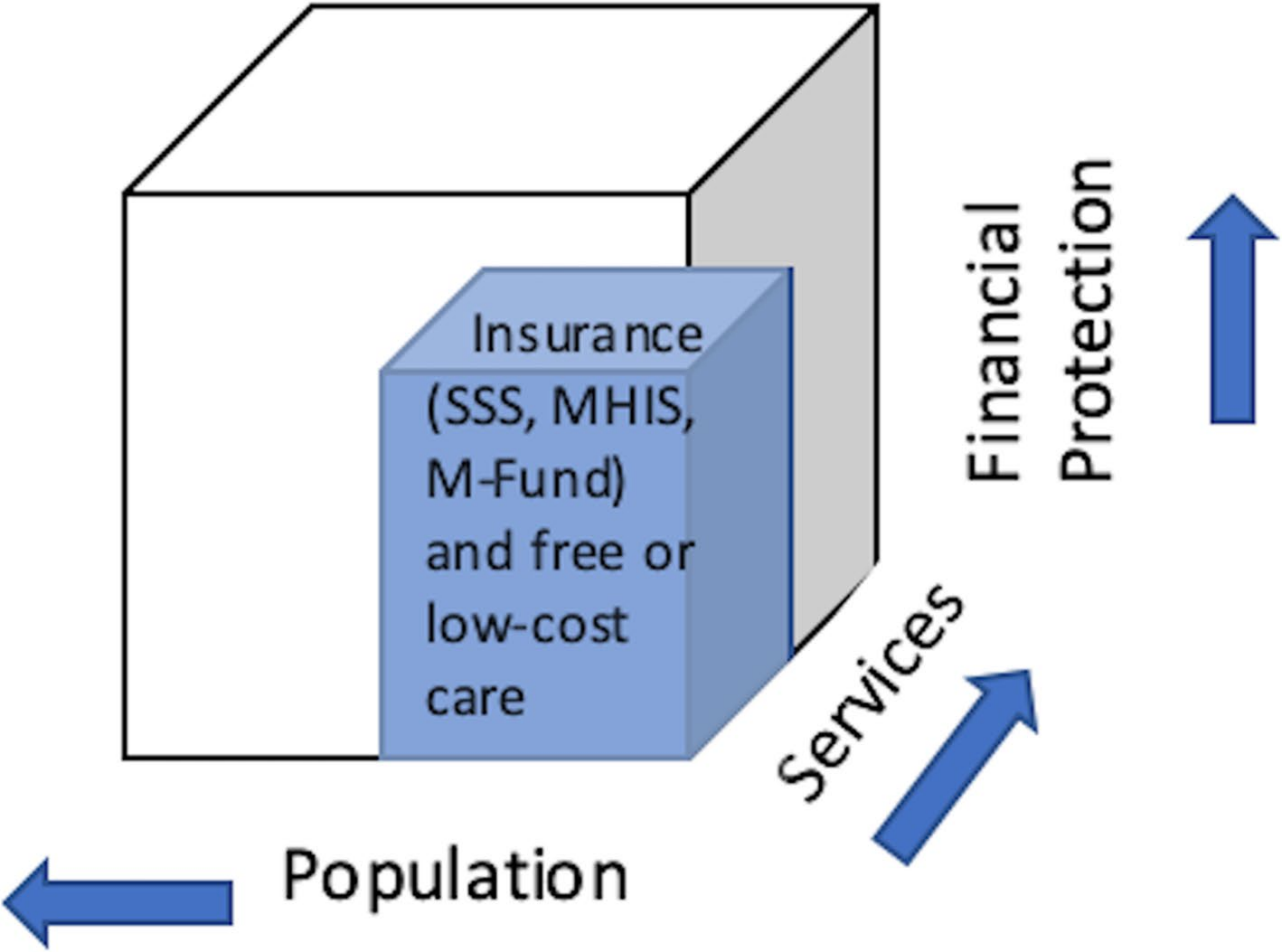
UHC cube for migrants’ antenatal and birth care in a Thai border region

We observe that together, the government and non-profit insurance schemes and free or low-cost services from NGO/non-profit providers have helped to increase population coverage of health services among migrants in this Thai border region and are especially valuable for undocumented individuals (see Fig. 1). Interviewees explained that combinations of insurance schemes and existing free or low-cost services have also increased the types of services that migrants would be able to receive without financial hardship. A clinician reported:

“If our patient has any M-Fund or health insurance care maybe we can send to the Thai hospital any time. Very easy for the staff and patient. Very easy. So, the challenge is if the patient has no health insurance, it will be very difficult (for the hospital and clinic staff) to communicate with each other”.

We found that the use of NGO/non-profit services in conjunction with an insurance scheme that gives access to government hospital services (M-FUND, MHIS, SSS), offers a robust possibility to get access to services from NGOs/non-profits in languages that migrants understand while also being eligible to get referred to a Thai government hospital for more complicated care. Myanmar and Thai language are substantially different and migrant workers have varying fluency in Thai language [21].

This hybrid healthcare provision by government and non-profit actors has also helped share the workload of migrant healthcare provision between organizations. NGOs/non-profits are most often the primary care providers for undocumented migrants. By providing low cost or free antenatal care and assistance for antenatal care and uncomplicated childbirth with linguistic and culturally appropriate services, NGO/non-profit clinics reduce the number of migrants that need treatment at the Thai government hospitals by only referring the “most difficult cases”. A healthcare provider from a Thai government hospital explained, “Instead of 100 cases we admit only ten cases because they (NGO and non-profit clinics) have been taking care of 90 cases”. Lack of antenatal care can result in major catastrophic complications in childbirth with greater morbidity and mortality for the mother and infant. In offering antenatal care and services for uncomplicated births, NGO/non-profit clinics are in effect triaging cases and limiting the burden of complicated cases for Thai government hospitals. While NGO/non-profit clinics and Thai government hospitals have different funding sources and treatment protocols, they are able to collaborate and transfer patient care between the non-profit and public institutions. Clinicians had also developed protocols to refer patients over the border to the nearest hospital in Myawaddy, Myanmar for planned caesarean section for high risk migrant patients.

The funding for migrant healthcare coverage in this border region comes from a variety of sources. Thai hospitals receive funds to provide care for migrants from a wide variety of sources including patient self-payment, SSS, MHI, MFUND and in some cases donations. NGO/non-profit clinics in this study were donor funded and received minimal or no funds from patients.

### Challenges to providing maternal health coverage for undocumented migrants

Providing maternal care to undocumented migrants who are not officially entitled to free healthcare from the state involves numerous challenges. Participants identified sustainability, debt, logistics and late arrivals as some of the difficulties they face as care providers.

#### Sustainability

Within this hybrid healthcare environment, where much of the primary ANC and uncomplicated birth care for undocumented migrants is provided by non-profit organizations, funding and associated sustainability is a challenge. Due to changes in the geo-political environment and associated development in nearby Myanmar, funders have reduced their support for healthcare organizations on the Thai side of the border. Participants spoke to the value of having these organizations provide uncomplicated birth care to migrants in the Thai border region and were concerned that funding shortages and subsequent reduction in services could overwhelm the healthcare system. One participant indicated that if NGO services were withdrawn, “This will have a huge effect on us. In each year, instead of 3,000 will become 6,000 patients come to us. Many of nurses will possibly resign”.

Participants from several Thai government hospitals indicated that their capacity was already stretched, and in some cases their intensive care unit beds were full. One individual explained that their hospital previously had an ANC outreach program for rural and remote areas that was not sustained for budgetary reasons.

#### Debt

Once undocumented migrants require secondary and tertiary care, such as caesarean sections and other emergency procedures during child birth, they must go to a government hospital either on the Thailand or Myanmar side of the border. Their decision will be influenced depending on which side of the border the emergency begins, proximity to the nearest hospital and the time of day. The official international border bridge that links Thailand to Myanmar closes every evening and reopens in the morning. Undocumented and uninsured migrant patients at government hospitals in Thailand will receive emergency birth care treatment, inclusive of caesarean sections, but in most cases would be subsequently billed for the services. Most patients can’t afford to pay the bills, leaving hospitals in debt. One participant estimated that the yearly debt of the five Thai border hospitals was 60 million baht (2 million USD). A clinician explains:

“For the service and standards, everyone receives the same thing. However, in terms of payment we have to follow the policy. In contrast it turns out that we cannot collect money from them…… As for the hospital view, I think this is quite a burden for us to bear, but we’re doing our best because this is our responsibility”.

Costs are especially high for critically ill patients who need to have emergency operations. Even a small number of cases that need obstetrical interventions can be very costly, particularly if the infant needs neonatal intensive care unit admission and extended care. This was emphasized by one participant, who referred to a case of a premature baby and a hospital stay of several months, with costs exceeding 100,000 baht (3163 USD). Another care provider who works at a hospital indicated that,

“In case of critical patients transferred to us who need to have emergency operations to give birth, the cost will be high. Even if we have a small number of cases the expenses of the Obstetric and Newborn sections will be the highest amount when compared with other sections of the hospital”.

For undocumented and uninsured patients, traditionally the organization who refers a patient pays for their care at the new health facility. This occurred for referrals between Thai government hospitals and referrals from NGO/non-profit clinics to Thai hospitals in the past. These costs can incur debt for organizations and in some cases influence decisions to refer patients. At the time of our research some organizations shifted their policies to no longer pay for referrals and suggested patients self-refer or go independently to the hospital.

#### Border region logistics

The geographical nature of the study area, a largely rural border region with one urban area, contributes to logistical challenges for healthcare provision.

For patients living in Myanmar, close to the Thailand border, due to geography and mountainous terrain and lack of roads in Myanmar it is easier to refer patients to Thailand than inland, however this would be an international referral and occur debt for the referring hospital. One participant, a health worker who supports women to get access to emergency birth care along the Thailand-Myanmar border, described the situation of a woman with eclampsia who went to Myawadee Hospital in Myanmar. The hospital had insufficient medicine and supplies and asked her if she wanted to go to the Thai hospital which would be more expensive or Kawkareik, a Myanmar hospital 50 km further inland. The woman died on her way to the Kawkareik hospital.

There are transportation difficulties for migrant women living in villages in more rural areas on the Thai side of the border as well as those in Myanmar. Care providers reported challenges getting patients to hospital for birth due to poor roads, nighttime border closures and lack of transportation in remote villages. A migrant woman may have attended ANC early, made her booking consultations at Myawaddy Hospital in Myanmar but finally if she has a problem in the night the most accessible hospital which provides EmOC may be in Thailand.

Logistical challenges extended to paperwork and complexities around expense reimbursement. Participants explained that patients who seek care at the hospital across the border in Myanmar have to go and buy supplies and do not receive receipts, making it difficult for NGOs to reimburse the patients for the costs. Treatment for birth services are officially free at government hospitals in Myanmar but patients must buy certain supplies at a shop outside the hospital. A clinician explains:

“The Myanmar health system follows the policy of free medical care. But in reality, if a pregnant woman goes and delivers in a hospital they will have to pay certain medical costs. They have to share the medical cost, like you mentioned buying the gloves or buying some antibiotics or sutures”.

#### Patients arrive late in critical condition

While care providers reported that most migrant patients typically had received ANC prior to giving birth, a small percentage of patients did not go to ANC and presented only during an emergency. These women needed complex emergency birth care, may have been in labour for a long time and presented to the clinic only as a last resort. Clinicians described the associated challenges of organizing a quick referral to the hospital and implementing lifesaving interventions. As the patients did not attend ANC, they also did not have the opportunity to register for migrant health insurance and as a result were uninsured.

“So last month we faced one high risk patient near term, near delivery. At that time, she went some clinic and they couldn’t provide any more care so that is why she came to our clinic. At that time she had never come to ANC at our clinic, and was a high risk patient, so at that time we definitely sent her to the hospital, in Thailand or Burma. At that time we had to pay for the operation cost. So we are really challenged by this situation.”

Interviewees from Thai government hospitals indicated that their institutions would provide emergency cesarean-sections and life saving measures to migrant patients regardless of their insurance status or ability to pre-pay. A clinician explains:

“When there is the emergency case, will you ask to see their nationality ID card before you help them? If you are not Thai we will not treat you. This is impossible. Not (only) the medical staff like us, general people will not ignore if someone is in danger”.

### The promise and limits of health insurance for undocumented migrants

The ultimate purpose of the M-FUND is to improve access for undocumented migrants that are not covered by the Thai government migrant health insurance program. Migrant women attending ANC at NGO/non-profit clinics were given the option to enroll and pay a 100 baht monthly fee for coverage.

The M-FUND was developed in part to help address financial concerns for organizations referring patients. By providing financial coverage, organizations would not be billed for costs associated with patients they refer. The fund was well received by participants affiliated with NGOs/non-profits and the Thai healthcare system and was identified as a mechanism that can help decrease institutional debt related to uninsured patients.

At the time of the data collection a large percentage of those enrolled were either pregnant or had a chronic condition. Some participants expressed concerns about the funds’ sustainability. One participant expressed:

“Today we are facing this challenge that we do not have enough income from the population to make this income sustainable. So we are depending on donors at the moment, voluntary contributions. Asking people to enroll in groups and giving decreasing premiums for group members”.

## Discussion

In this article we present a case study of improving undocumented migrants’ inclusion in universal health coverage. Along the Thailand-Myanmar border NGOs/non-profits and Thai healthcare providers have collaborated to create an environment where undocumented migrants can access free or low-cost ANC. Birth services are free or low cost at migrant clinics but undocumented migrants without health insurance can be billed for emergency care if they need to present at a Thai hospital. There is a mutual interest in and engagement with providing care to undocumented pregnant migrants. NGO/non-profit clinics have helped to reduce the burden of providing antenatal and uncomplicated birth care from the Thai government hospitals and play an important role in sustaining maternal care for this population. It is possible that the provision of maternal healthcare by non-profit and NGO actors may result in an underestimation of the extent of healthcare services needed and could hinder government incentive to expand and provide more comprehensive healthcare to undocumented pregnant migrants. Collaboration and communication between government and non-government sectors is important to help identify the true needs of undocumented migrants and plan for better care provision.

From this research we know that Thai government hospitals will normally provide emergency treatment to migrant women who need a caesarean section during childbirth, irrespective of their ability to pay. However, official policy states that uninsured migrants must be billed for services. Providers who participated in our study also voiced concerns about hospital debt from unpaid bills and associated implications for sustainability. Our results echo a review of providers’ challenges in caring for migrant patients that cited resource limitations and dissonance between laws and professional codes as some of the difficulties clinicians experience [22].

Our research took place in an international border region and other challenges, beyond health care entitlements and funding challenges, complicated care provisions. Night time border closures, poor roads, lack of transport may contribute to late arrivals and complex care needs. We anticipate that other low-and-middle income country border regions migrants may experience similar challenges, which points to the need to improve health coverage on both sides of international borders and to implement supports to help patients get to the clinic when they are in labour.

In this Thailand-Myanmar border region a voluntary non profit migrant health insurance was put in place and helped address both patient’s catastrophic healthcare costs and the financial sustainability of healthcare providing institutions. Voluntary migrant health insurance helped give patients who previously had no entitlements to government hospital services, free emergency birth services when needed. While this model begins to approach universal coverage, whereby people can access the care they need without financial hardship, the voluntary nature of the M-FUND means that some patients will slip through the cracks. Those who didn’t enroll will not have coverage. Of specific concern are late arrivals, women who did not seek ANC and present during an emergency.

This case study provides several lessons for policy makers which are relevant to improving health coverage for undocumented migrants in the international context. Firstly, a non-profit insurance fund for undocumented population can help to improve healthcare entitlements, provide financial protection and reduce service providers’ debt. To our knowledge non-state, non-profit health insurance programs for migrants are novel, and the M-FUND is among the first to be implemented. Secondly, there can be critical limits to voluntary programs for undocumented migrants. High costs associated with emergency caesarean section and other high-cost procedures along with gaps in insurance coverage challenge the sustainability for both NGOs/non-profits and government health providers and may cause financial catastrophe for patients. We observe that mechanisms to provide coverage without enrollment, through tax funding or other means may help ensure coverage for all. Thirdly, in international border regions with high mobility, it may be important to consider cross border referrals for migrant patients, as well as improvements to coverage on both sides of the border. Strengthened cross border coverage can help migrants get the care they need, when they need it and without financial hardship.

Our study has several limitations. As our research took place on the Thailand side of the border, we did not have an opportunity to interview clinicians working in nearby Myanmar hospitals and thus do not have a full sense of the challenges they experience. We chose in this paper to highlight the perspectives of key informants who work to provide care to migrants and have thus not integrated the perspectives of undocumented migrant who were accessing maternal healthcare. While this paper presents the care provider perspective on including migrants in universal health coverage, we have published a manuscript based on patient perspectives that addresses care decision making when choosing where to give birth [23]. Future studies on inclusion of migrants in UHC in border areas may be enhanced by including a data collection on both sides of the international border.

## Conclusions

Coverage for undocumented migrants remains a challenge for UHC and to date there are limited academic studies from LMICs. Additionally, UHC is often discussed at the policy level but we have not located any maternal specific case studies at the local level of the inclusion of migrants which incorporate care providers’ perspective. This study contributes to the literature by providing a local LMIC case study of migrant maternal healthcare coverage along with concrete examples from clinicians on the challenges of implementing coverage and providing care in this context. We hope that it will be useful for other LMIC jurisdictions which are considering interventions to improve health coverage for undocumented migrants.

## Supplementary Information

**Additional file 1.**

## Data Availability

Our data is qualitative and contains identifying information. Public availability of data would compromise patient confidentiality and privacy. Sharing excerpts of transcripts would violate the agreement to which patients and key informants consented when they agreed to participate. For these reasons, data cannot be shared.

## Abbreviations

ANC: Antenatal Care
EmOC: Emergency Obstetric Care in Labor
UHC: Universal Health Coverage
LMIC: Low and Middle-Income Countries
SSS: Social Security Scheme
MHIS: Migrant Health Insurance Scheme
M-FUND: Migrant Fund
