# Supplementary material for: Including undocumented migrants in universal health coverage: a maternal health case study from the Thailand-Myanmar border
Source: BMC Health Serv Res. 2021 Dec 7;21:1315. doi: 10.1186/s12913-021-07325-z (PMC8650330; doi:10.1186/s12913-021-07325-z)
Supplement: Supplementary file 1 — Additional file 1. [file 12913_2021_7325_MOESM1_ESM.docx]

**Supplementary file 1: Key Informants interview guides**

Inquire about: locations of emergency birth services, service entitlements, insurance, referrals, health system capacity and sustainability. The interviewer ask participants the prompting questions such as:

- Please describe your role in the organization where you work.
- Which target populations does your organization work with?
- Does your organization provide treatment?
  - Antenatal care?
  - Birth care with the assistance of a trained midwife?
  - Emergency obstetrical care?
- Do patients need to pay out of pocket for services?
  - If yes please describe
- If your organization does not provide treatment for some of these conditions, where are patients referred to?
- Where are other ANC care and emergency birth services for migrant women located in this area?
- Are there specific barriers to quality ANC and emergency birth services in this region?
- In your opinion, are there sufficient emergency care options for migrant women? Are the care options sustainable?
- From your experience what types of barriers do non-Thai migrant women face when accessing ANC care and emergency birth services?
  - Probes: self perceived need, gender responsibilities, legal status, language, pressure to work, treatment cost, out of pocket payments, low health knowledge.
- How does legal status and associated entitlements influence migrant women’s ability to access emergency birth services?
- Does having health insurance impact migrant women’s access to antenatal and birth care?
- In the last two years, has the organization you work with changed the care for pregnant women is delivered?
  - (If yes) How?
  - (If yes) What are the reasons for these changes?
- Inyour opinion, what types of further improvements or adaptations could help to improve access to emergency obstetrical care among your target population(s)?
- Are women routinely counseled on family planning after they give birth?
- Do treatment providers suggest family planning options to avoid future pregnancy based on what happened in pregnancy or delivery?
